# Identification of hub genes and potential ceRNA networks of diabetic cardiomyopathy

**DOI:** 10.1038/s41598-023-37378-5

**Published:** 2023-06-24

**Authors:** Jun Hou, Wan Yi Liang, Shiqiang Xiong, Pan Long, Tian Yue, Xudong Wen, Tianchen Wang, Haoyu Deng

**Affiliations:** 1 Department of Cardiology, The Third People’s Hospital of Chengdu/Affiliated Hospital of Southwest Jiaotong University, Chengdu, Sichuan China; 2 School of Life Science and Engineering, Southwest Jiaotong University, Chengdu, Sichuan China; 3 Department of Microbiology and Immunology, Faculty of Science, University of British Columbia, Vancouver, BC Canada; 4 Department of Gastroenterology and Hepatology, Chengdu First People’s Hospital, Chengdu, Sichuan China; 5 Alfred E. Mann Department of Biomedical Engineering, University of South California, Los Angeles, CA USA; 6 Department of Medicine, Faculty of Medicine, University of British Columbia, Vancouver, BC Canada; 7 Centre for Heart and Lung Innovation, St. Paul’s Hospital, University of British Columbia, 1081 Burrard Street, Vancouver, BC V6Z 1Y6 Canada

**Keywords:** Disease genetics, Cardiomyopathies, Long non-coding RNAs, miRNAs, Computational biology and bioinformatics, Genetics

## Abstract

Diabetic cardiomyopathy (DCM), a common complication of diabetes, is defined as ventricular dysfunction in the absence of underlying heart disease. Noncoding RNAs (ncRNAs), including long noncoding RNAs (lncRNAs) and microRNAs (miRNAs), play a crucial role in the development of DCM. Weighted Gene Co-Expression Network Analysis (WGCNA) was used to identify key modules in DCM-related pathways. DCM-related miRNA-mRNA network and DCM-related ceRNA network were constructed by miRNA-seq to identify hub genes in these modules. We identified five hub genes that are associated with the onset of DCM, including Troponin C1 (Tnnc1), Phospholamban (Pln), Fatty acid binding proteins 3 (Fabp3), Popeye domain containing 2 (Popdc2), and Tripartite Motif-containing Protein 63 (Trim63). miRNAs that target the hub genes were mainly involved in TGF-β and Wnt signaling pathways. GO BP enrichment analysis found these miRNAs were involved in the signaling of TGF-β and glucose homeostasis. Q-PCR results found the gene expressions of Pln, Fabp3, Trim63, Tnnc1, and Popdc2 were significantly increased in DCM. Our study identified five hub genes (Tnnc1, Pln, Fabp3, Popdc2, Trim63) whose associated ceRNA networks are responsible for the onset of DCM.

## Introduction

Diabetic cardiomyopathy (DCM) is clinically defined as the existence of abnormal myocardial structure and performance in the absence of other cardiac diseases, including coronary artery disease, hypertension, and significant valvular diseases, in individuals with diabetes mellitus^1^. It is established that the prevalence of heart failure in diabetic patients ranges from 19 to 26% worldwide^2^. Clinically, DCM is usually asymptomatic in the early stages of its evolution through left ventricle (LV) hypertrophy and decreased LV compliance, which are characterized by impaired early diastolic filling. Subsequently, LV dilation and symptomatic heart failure occur after the development of systolic dysfunction^3^. Unfortunately, screening approaches including B-type natriuretic peptide, exercise stress testing, and echocardiography, do not seem to be sufficiently sensitive in identifying subclinical dysfunction in diabetic patients^4^. As a result, ongoing studies are vital to understanding the precise mechanisms that initiate and progress DCM and to developing novel strategies to reduce the risk of heart failure in diabetic patients.

Noncoding RNAs, including long noncoding RNAs (lncRNAs) and microRNA (miRNA), play an important role in many biological processes and diseases including diabetes and its complications^5–7^. It has been shown that diverse RNA molecules harboring miRNA Recognition Elements (MREs) can act as competing endogenous RNAs (ceRNAs) against the common pool of miRNAs for communication, engendering the ceRNA hypothesis^8,9^. More recently, a growing body of evidence has also found that ceRNA activity of lncRNAs can serve as natural miRNA decoys in human development and pathophysiological conditions^10,11^. The ceRNA hypothesis plays an impactful role in diabetes and diabetic complications. For example, Zhou et al.^12^ reported that lncRNA myocardial infarction–associated transcript (MIAT) functions as a competing endogenous RNA to up-regulate DAPK2 by sponging miR-22-3p in DCM; Feng et al.^13^ found that lncRNA DCM-related factor (DCRF) regulates cardiomyocyte autophagy by targeting miR-551b-5p in DCM; Yang et al.^14^ demonstrated that lncRNA, Kcnq1ot1, is over-expressed in DCM, and silencing Kcnq1ot1 inhibits pyroptosis by regulating miR-214-3p and caspase-1 expressions. In addition, a recent study^15^ showed that lncRNA, ZFAS1, acts as a ceRNA to sponge miR-150-5p and down-regulate CCND2, consequently promoting cardiomyocyte ferroptosis and DCM development. These studies indicate that noncoding RNA through the ceRNA hypothesis may play an important role in DCM.

**Figure 1:**
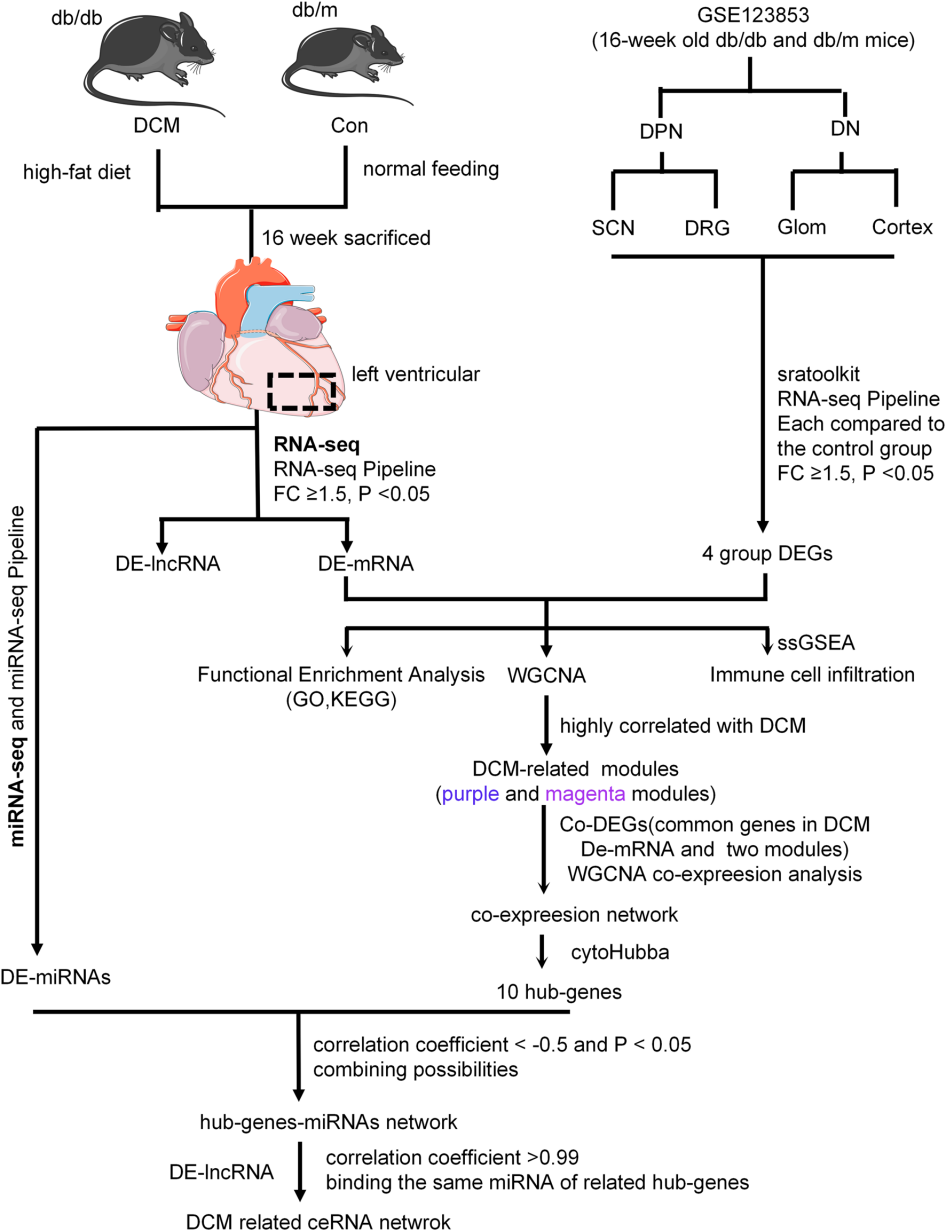
Flowchart describing the schematic overview of the study design. DPN, diabetic peripheral neuropathy, DN, diabetic nephropathy.

In this study, we performed RNA-seq and miRNA sequencing on heart tissue from db/db mice to explore the transcriptome alterations in DCM pathogenesis, including lncRNA, miRNA, and mRNA. We further analyzed possible mechanisms by which altered immune cell infiltration may trigger DCM. This study provides a potential novel understanding of the pathogenesis of DCM and proposes several potential therapeutic targets. The research design is shown in Fig. 1.

## Materials and methods

### Animals and tissue samples

All procedures were performed under the guidelines for animal care and approved by the Animal Care and Ethics Committee of Southwest Jiaotong University. The present study was reported in accordance with the ARRIVE 2.0 Essential 10 guidelines. Two murine strains with C57BL/6 background including db/m and db/db mice were used as the spontaneous diabetic cardiomyopathy model and healthy control model, respectively. Three newborn healthy male db/m mice and three newborn healthy male db/db mice were purchased from Shanghai Nanfang Model Biotechnology Co. LTD (Shanghai, China). Db/m mice were given ad libitum access to tap water and laboratory feed for 16 weeks. Db/db mice were fed a high-fat diet for 16 weeks. The ultrasound system (VisualSonics, Toronto, ON, Canada) was used for echocardiographic measurements. After anesthetic treatment with avertin, the mice were shaved to expose the chest area and fixed onto a flat plate. Left ventricular systolic diameter (LVDs), LV diastolic diameter (LVDd), LV ejection fraction (EF), and LV fractional shortening (FS) were determined and averaged from 6 consecutive cardiac cycles^16^.

### Data preparation

The data set, GSE123853, was retrieved and downloaded from the Gene Expression Omnibus (GEO) database^17^. This dataset was employed to identify distinct molecular pathways regulating diabetic peripheral neuropathy (DPN) and nephropathy (DN). This dataset also included RNA-seq data of four diabetic complication-prone tissues (sciatic nerve (SCN), dorsal root ganglia (DRG), kidney glomeruli (Glom), and kidney cortex (Cortex)). Raw data of GSE123853 were downloaded from the Sequence Read Achieve (SRA) with accession SRP173448 and subsequently converted to FASTQ with sratoolkit^18^.

### RNA-Seq pipeline

RNA was isolated from the left ventricle of three db/db mice and three db/m mice, respectively. RNA was used to generate barcoded complementary DNA (cDNA) libraries using the NEBNext Ultra RNA library prep with rRNA depletion (New England Biolabs). Indexed libraries were sequenced in 2 × 150 bp configuration on the Illumina HiSEQ platform. Briefly, first-strand and second-strand cDNA were synthesized from the input RNA, and single primer isothermal amplification (SPIA) of the resultant cDNA was performed, followed by mechanical shearing of double-stranded cDNA (ds-cDNA) (Covaris E220 ultrasonicator; Covaris Inc, MA). Subsequently, end repair and A-tailing of sheared cDNA and construction of unique barcoded libraries by addition of adapters and PCR amplification (8 cycles) were conducted. The Agencourt AmPure XP bead (Beckmann Coulter) purified libraries were quantified by quantitative PCR (q-PCR) and size distribution was checked using the Agilent TapeStation 2200 system. The libraries were subjected to paired-end 150 bp sequencing on HiSeq 2500 sequencing system (Illumina). For all samples, including public data, paired-end reads were mapped to the mm 10 genome assembly using RNA-seq aligner, STAR, with default parameters. Duplicate and multi-mapped reads were removed with samtools^19^. Following alignment, reads were counted per gene and fragments, per kilobase per million (FPKM), with featureCounts tool and GENCODE m28 and NONCODE v6 annotation^20,21^. Gene counts were then normalized and analyzed for identified differential expression mRNAs (DE-mRNAs) and lncRNAs (DE-lncRNAs) using the Bioconductor package edger, which is operated with a > 1.5-fold change and P-value < 0.05 cut-offs^22^**.** FPKM was used for Weighted Gene Co-Expression Network Analysis (WGCNA).

### miRNA-seq pipeline

miRNA was isolated from the left ventricle (3 db/db mice and 3 db/m mice). The libraries were generated using NEBNext®Multiplex Small RNA Library Prep Set for Illumina® (NEB, USA). After library preparation and pooling of different samples, the samples were subjected to Illumina NextSeq500 sequencing. Raw data with fastq format containing N, low quality, 5’ adapter contaminants, ploy A, and low-quality reads were removed from the reads. Raw data without a 3’adapter or the insert tag was also removed from the reads as well. The small RNA tags from rRNA, tRNA, small nuclear RNA (snRNA), and small nucleolar RNA (snoRNA) were detected by mapping with the Rfam database and further mapped to the reference genome, mm10, by Bowtie software^23^. Known miRNA was identified by mapping to the miRBase database with R package, miRDeep2^24^. MiRDeep2 was also used to count the reads numbers mapped to each miRNA. R package edgeR was used for differential expression analysis. A cut-off with fold change ≥ 1.5 and P-value < 0.05 was used for identifying differential expression miRNAs (DE-miRNAs).

### WGCNA

We used the WGCNA package (version 1.60) in R to find and combine highly correlated mRNAs into mRNA modules, which permitted an examination for correlation between each module and diabetic complication-prone^25^. Before using the WGCNA, we used surrogate variable analysis (sva) package and object-oriented microarray and proteomics analysis (oompaBase) package to remove the batch effect between different samples^26^. The power of β = 5 (scale-free R^2^ = 0.8) was selected as the soft threshold to ensure a scale-free network (Figure S1). The dynamic tree cutting method was used to cluster the mRNAs in layers, using 50 as a minimum size cutoff, and the cut height = 0.3 was applied to merge highly similar modules. Different mRNA modules were labeled with varying colors; the gray module represents mRNAs that could not be merged^27^. Pearson correlation analysis was implemented to evaluate the correlation between mRNAs and susceptibility to diabetic complications.

### Functional enrichment analysis

Gene Ontology (GO), including cellular components, molecular function, biological process, and Kyoto Encyclopedia of Genes and Genomes (KEGG) pathway^28,29^, were analyzed using the R package clusterProfiler (version 3.2.14), as described previously^30^. Gene set enrichment analysis (GSEA) was performed using KEGG pathway annotation data, analyzed with the package clusterProfiler, and displayed on ridgeline plot^31^. Cell senescence related genes were download from CSGene database^32^. Heart regeneration-related genes were download from Regeneration Roadmap^33^. DE-lncRNAs have a high positive correlation (correlation coefficient > 0.9) with cell senescence-related genes and heart regeneration-related genes, identifying potential cell senescence-related or heart regeneration-related lncRNAs.

### Immune cell infiltration

The immune cell infiltration status was acquired from bulk RNA-sequencing data by applying the single-sample gene set enrichment approach to the transcriptomes based on single-sample GSEA (ssGSEA), and ultimately acquired through the R package, gene set variation analysis (GSVA)^34^ (Table S1.). A total of 25 immune cells were used in this study, which include activated B cell, activated CD4^+^ T cell, activated CD8^+^ T cell, central memory CD4^+^ T cell, central memory CD8^+^ T cell, effector memory CD4^+^ T cell, effector memory CD8^+^ T cell, immature B cell, memory B cell, T follicular helper cell, Tγδ, Th1, Th2, Th17, Treg, natural killer (NK) cell, eosinophil, activated dendritic cell (DC), immature DC, neutrophil, plasmacytoid DC, macrophage, mast cell, monocyte, and NKT cell^35^.

### Prediction of target miRNAs and construction of ceRNA networks

According to the ceRNA hypothesis, miRNAs can induce gene silencing and down-regulate gene expression by binding to mRNA, and lncRNAs that are rich in miRNA binding sites can act as a miRNA sponge, leading to changes in expression levels of miRNA-target mRNAs. We first identified differentially expressed miRNAs (DE-miRNAs) in DCM and then calculated the Pearson correlation coefficient between these DE-miRNAs and hub genes. DE-miRNAs paired with correlation coefficient < -0.5 and P < 0.05 were considered as potential miRNAs that can down-regulate the expression of the target hub gene. The ability to bind target hub gene was established by RNAhybrid (energy threshold = -20, G: U in seed)^36,37^. Furthermore, DE-lncRNAs that have a high positive correlation coefficient (correlation coefficient > 0.99) with hub genes that bound the same miRNAs were used to construct ceRNA networks. The result was presented as a mRNA-miRNA co-expressed network and ceRNA networks by Cytoscape^38^.

### qRT-PCR assay

Transcript One-Step gDNA Removal (YEASEN, shanghai, China) and cDNA Synthesis SuperMix (YEASEN, shanghai, China) were employed during the reverse transcription process according to the manufacturer’s instructions. cDNA was amplified with SYBR q-PCR Master Mix (EnzyArtisan, shanghai, China) in StepOnePlus (Applied Biosystems) equipment. The 2-ΔΔCt method was used to calculate the relative lncRNA expression, and each hole was repeated three times to ensure quantitative accuracy. The sequences of primers used for qRT-PCR are listed in Table S2, and GAPDH was used as an internal reference gene.

### Statistics analysis

Statistical analyses were accomplished using R. The student’s t-test was used for comparison between two independent groups. A P < 0.05 was considered statistically significant.

### Ethics approval and consent to participate

All animal experiments were performed according to procedures approved by the Laboratory Animal Ethics Committee of Southwest Jiaotong University.

## Results

### DCM model evaluation

Representative echocardiographic images were selected to display the impairment of cardiac function in DCM (Figure S2A). Echocardiographic data show that the ejection fraction (EF%) was significantly decreased in db/db mice (37.31 ± 1.84%), as compared with db/m counterparts (85.60% ± 3.57%). Similarly, fraction shortening (FS%) was also markedly reduced in db/db (26.28% ± 1.42%) than in db/m (47.73% ± 1.81%) mice, suggesting that cardiac function was impaired in DCM (Figure S2B).

### Gene expression analysis of DCM

**Figure 2:**
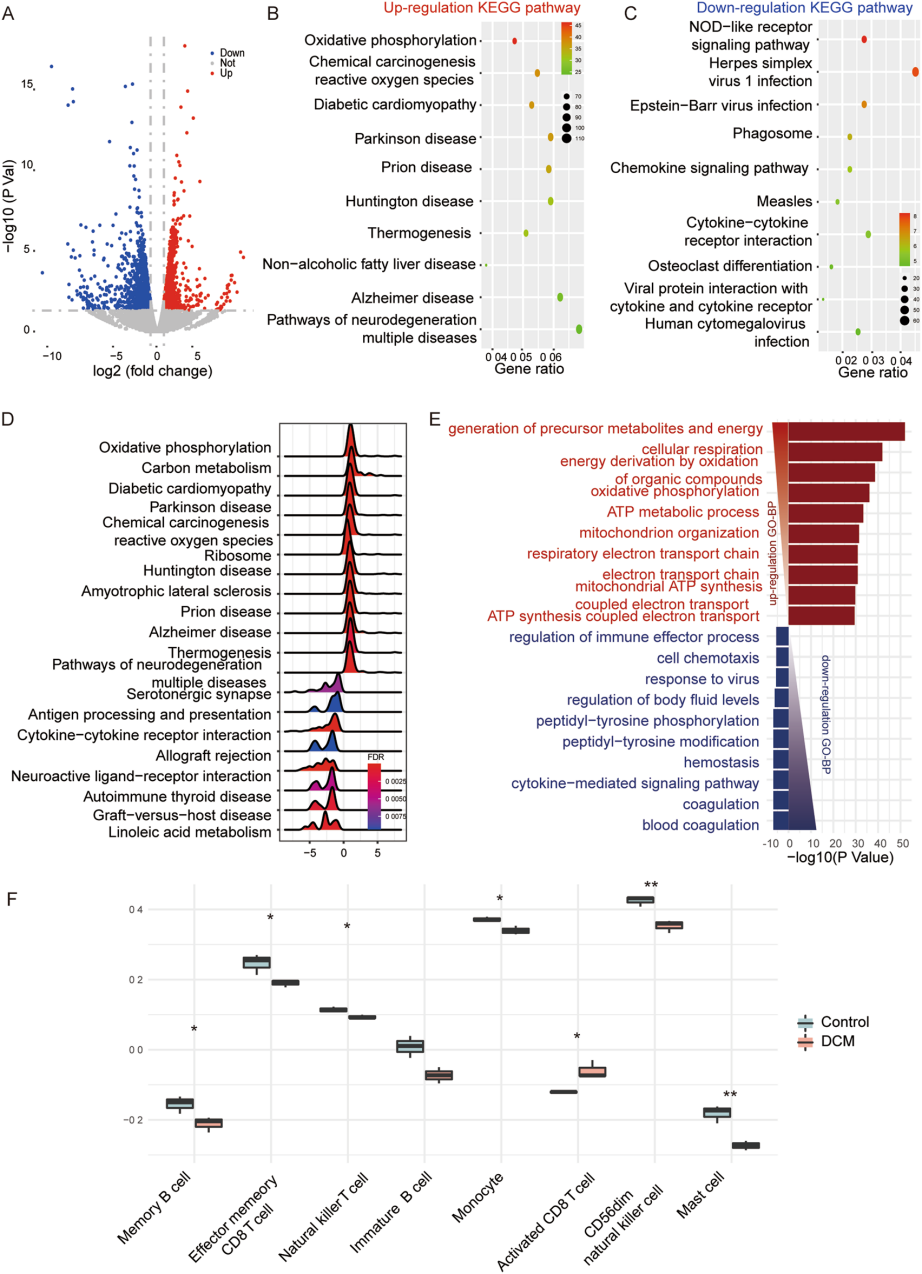
Screening of differentially expressed genes in DCM. (**A**) Volcano of the DCM. (**B**,**C**) Results of the KEGG pathway analysis of the up-regulation DEGs and down-regulation DEGs. The colors indicate the significance [− log10(P-value)], and the size of the circles represents the number of genes enriched in the corresponding annotation. (**D**) Ridge plots of the results of the GSEA analysis. (**E**) GO BP enrichment analysis of the DEGs. Up-regulated GO BP is shown with horizontal axis > 0, and down-regulated GO BP is shown with horizontal axis < 0, respectively. The size of the horizontal axis is set to − log10(P value). (**F**) Boxplot showed the different immune cell infiltration in DCM and control. *P < 0.05; **P < 0.01.

To distinguish expressed genes between DCM and healthy controls, we performed RNA-seq experiments using db/db mice model and its corresponding control db/m model. Our results reflect that 1493 genes were up-regulated and 1609 genes were down-regulated in DCM mice compared with control mice (Fold Change ≥ 1.5, P < 0.05) (Fig. 2A and Table S3). We performed the KEGG pathway analysis to determine possible regulation mechanisms of these differentially expressed genes (DEGs) in the process of DCM. The top 10 pathways that involved up-regulated DEGs included oxidative phosphorylation and reactive oxygen species (Fig. 2B). On the other hand, the top 10 pathways associated with down-regulated DEGs included NOD-like receptor signaling pathway (Fig. 2C). Similar results were obtained by performing GSEA of KEGG pathway. The results reveal that the genes associated with oxidative phosphorylation and reactive oxygen species were mainly up-regulated in DCM mice (Fig. 2D). GO analysis found up-regulated DEGs were associated with some biological processes, which include ATP metabolic process, mitochondrion organization, and mitochondrial ATP synthesis, while down-regulated DEGs were associated with biological processes including the regulation of immune effector (Fig. 2E). Besides, the analysis also found abnormal expression in an abundant of genes associated with senescence and heart regeneration in db/db mice (Figure S3).

Since DCM can be facilitated by alterations in both adaptive and innate immune systems, we further examined the difference of immune cell infiltration in left ventricle between DCM mice model and control model. The results reveal that activated CD8 + T cells infiltration was significantly increased in the DCM compared to control, while the infiltration of memory B cells, NKT natural killer cells, monocytes, and mast cells was significantly decreased, indicating an impaired profile of innate immunity in DCM (Fig. 2F).

### The difference of KEGG pathway and immune cell infiltration between DCM and other diabetic complications

**Figure 3:**
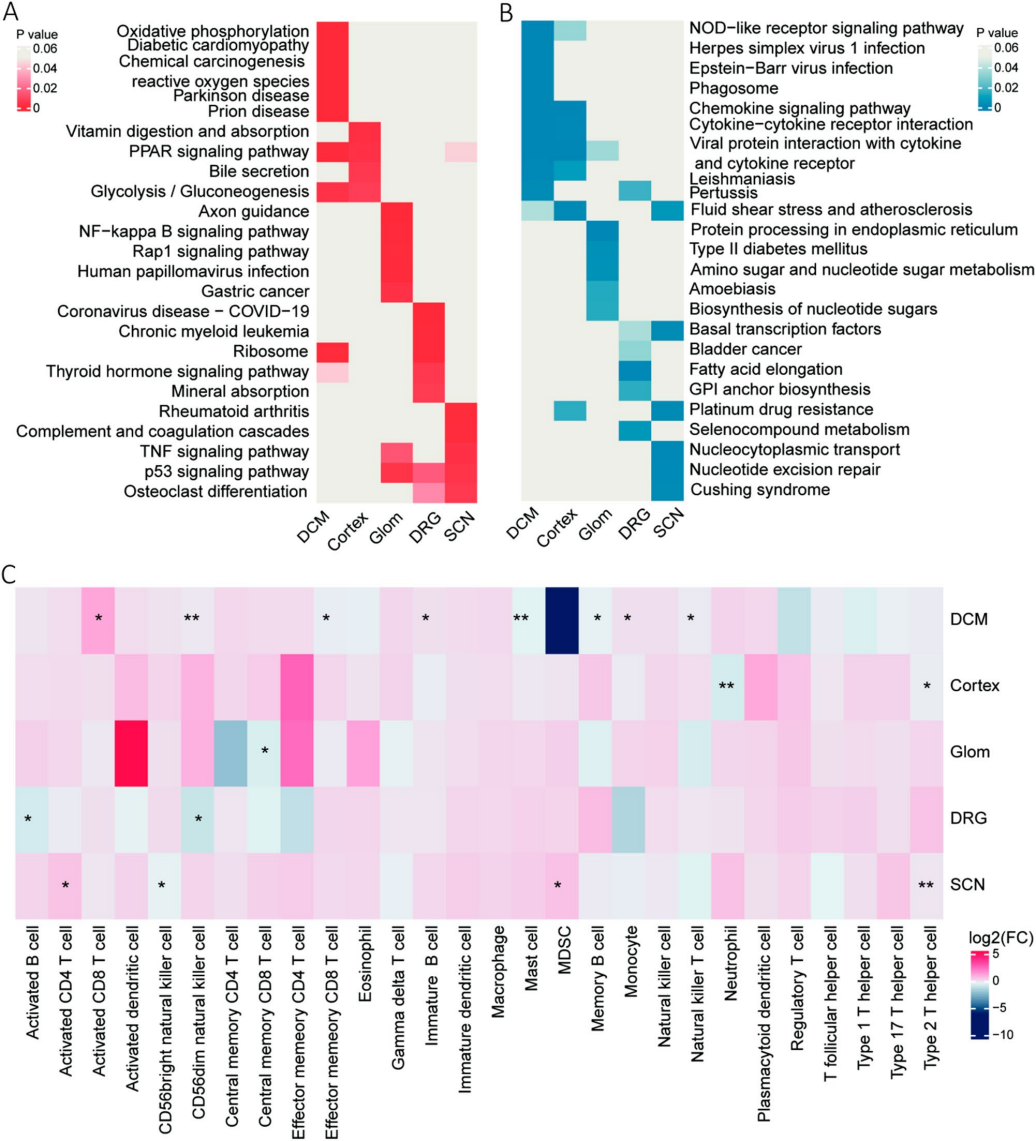
Compare the differences among different diabetic complications. (**A**) Heatmap showed the p-value of the top 5 up-regulated KEGG pathway from different diabetic complications. (**B**) Heatmap showed the p-value of the top 5 down-regulated KEGG pathway from different diabetic complications. (**C**) Heatmap showed the fold change of immune cell infiltration in different diabetic complication groups, compared to control. *P < 0.05; **P < 0.01.

At present, most studies have focused on a specific diabetic complication. For example, Li et al. have identified the hub genes of diabetic nephropathy. Yet, few studies have compared the potential pathogenesis of hyperglycemia-induced diabetic complications among different organs. Hence, in order to compare the potential causative roles of dysregulated genes between DCM and other diabetic complications, including diabetic peripheral neuropathy (DPN, including SCN and DRG) and diabetic nephropathy (DN, including Glom and Cortex), we determined the different gene expression files in each diabetic complication by comparing with respective health control organs. Moreover, we performed enrichment analysis to identify the difference in biological processes among five tissues from diabetic complications that differed based on DEGs. The results demonstrate that up-regulated DEGs in DCM were enriched in multiple signaling pathways, including oxidative phosphorylation and reactive oxygen metabolism, as compared to other diabetic complications (Fig. 3A). In addition, we found that down-regulated DEGs in DCM were associated with NOD-like receptor signaling and chemokine signaling, while down-regulated DEGs in other diabetic complications mainly focused on other pathways, such as fluid shear stress, atherosclerosis, nucleocytoplasmic transport, and nucleotide excision repairment (Fig. 3B). Given the discovery that down-regulated DEGs in DCM is highly associated with the host innate immunity pathway, we further performed immune cell infiltration analysis to determine specific immune cell subtypes. The results show that the infiltration of activated CD8^+^ T cells was significantly increased in DCM, as compared to other diabetic complications (Fig. 3C). This indicates that excessive specific immune cell infiltration within the heart may contribute to the development of DCM.

### Identification of key module promoting DCM development

**Figure 4:**
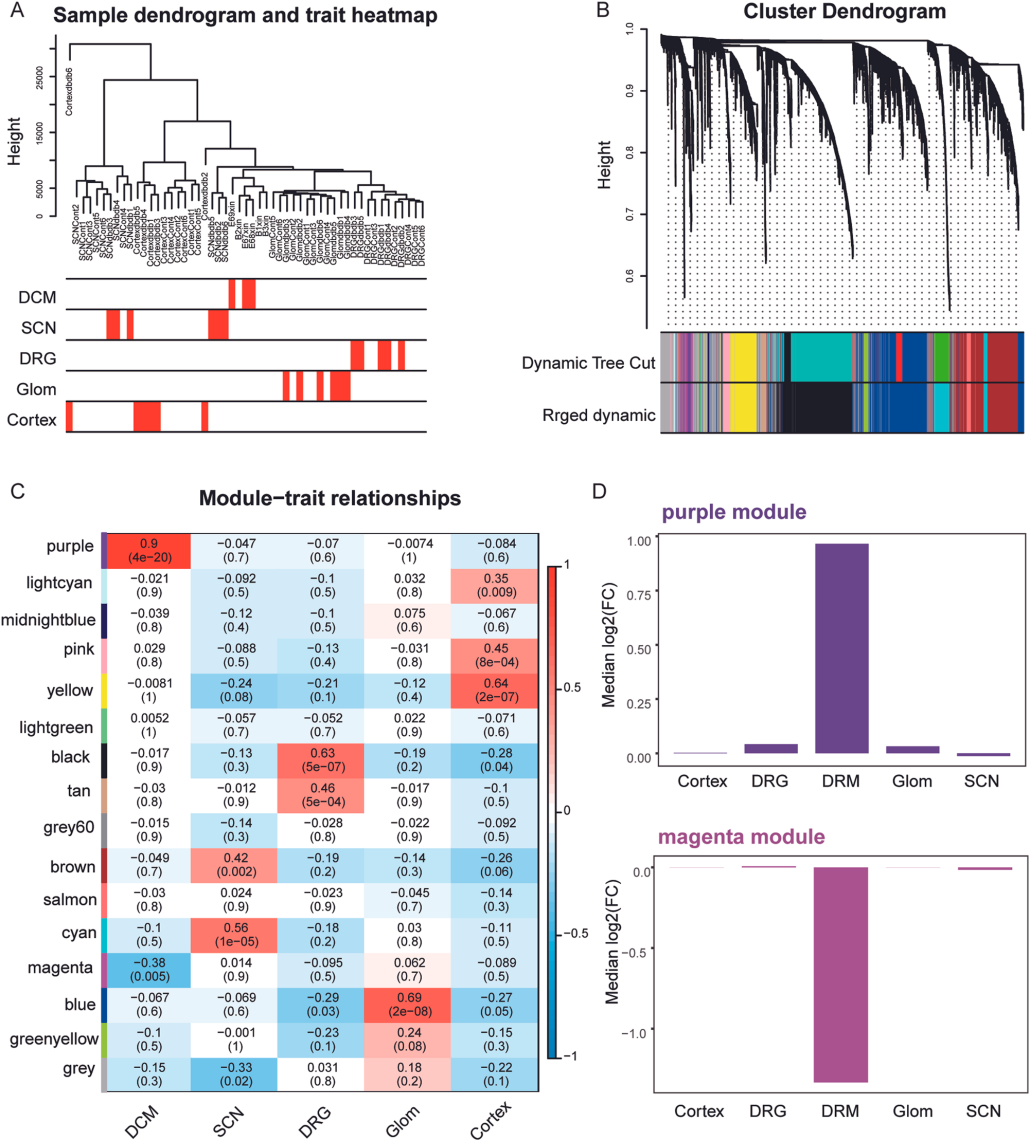
Identified DCM-related module. (**A**) Cluster dendrogram of samples based on their Euclidean distance. Sample dendrogram and trait heatmap. (**B**) Cluster dendrogram of co-expression network modules with dissimilarities based on topological overlap, in addition to assigned module colors. Top: cluster dendrogram shows the result of hierarchical clustering with each line representing one gene. Bottom: colored row below the dendrogram indicates the module membership identified. Different colors represent different co-expression network modules for the significant genes. (**C**) Module-trait relationships. Each row represents a color module (normal and diabetic nephropathy) and every column represents a clinical trait (DCM, SCN, DRG, Glom, and Cortex), respectively. Each cell contains the corresponding value of correlation in the first line and p-value in the second line, respectively. The cell color presents the correlation according to the color legend. (**D**) Median fold change of genes from the purple module (top) and the magenta module (bottom) in different diabetic complication groups, compared to control.

Next, we performed WGCNA to further determine the key factors regulating DCM development. The sample cluster tree was constructed and demonstrated in Fig. 4A. Through WCNA analysis, 15 co-expression modules were constructed (Fig. 4B). Module-trait relationship analysis revealed that purple modules were positively correlated to DCM, while the magenta module was negatively associated with DCM (Fig. 4C). In addition, gene expression in the purple module was significantly up-regulated in DCM compared to other diabetic complications. Contrarily, gene expression in the magenta module was significantly down-regulated in DCM compared to other diabetic complications (Fig. 4D). Altogether, these results suggest that genes in the purple and magenta module may affect DCM development.

### Functional enrichment analysis of Co-DEGs

**Figure 5:**
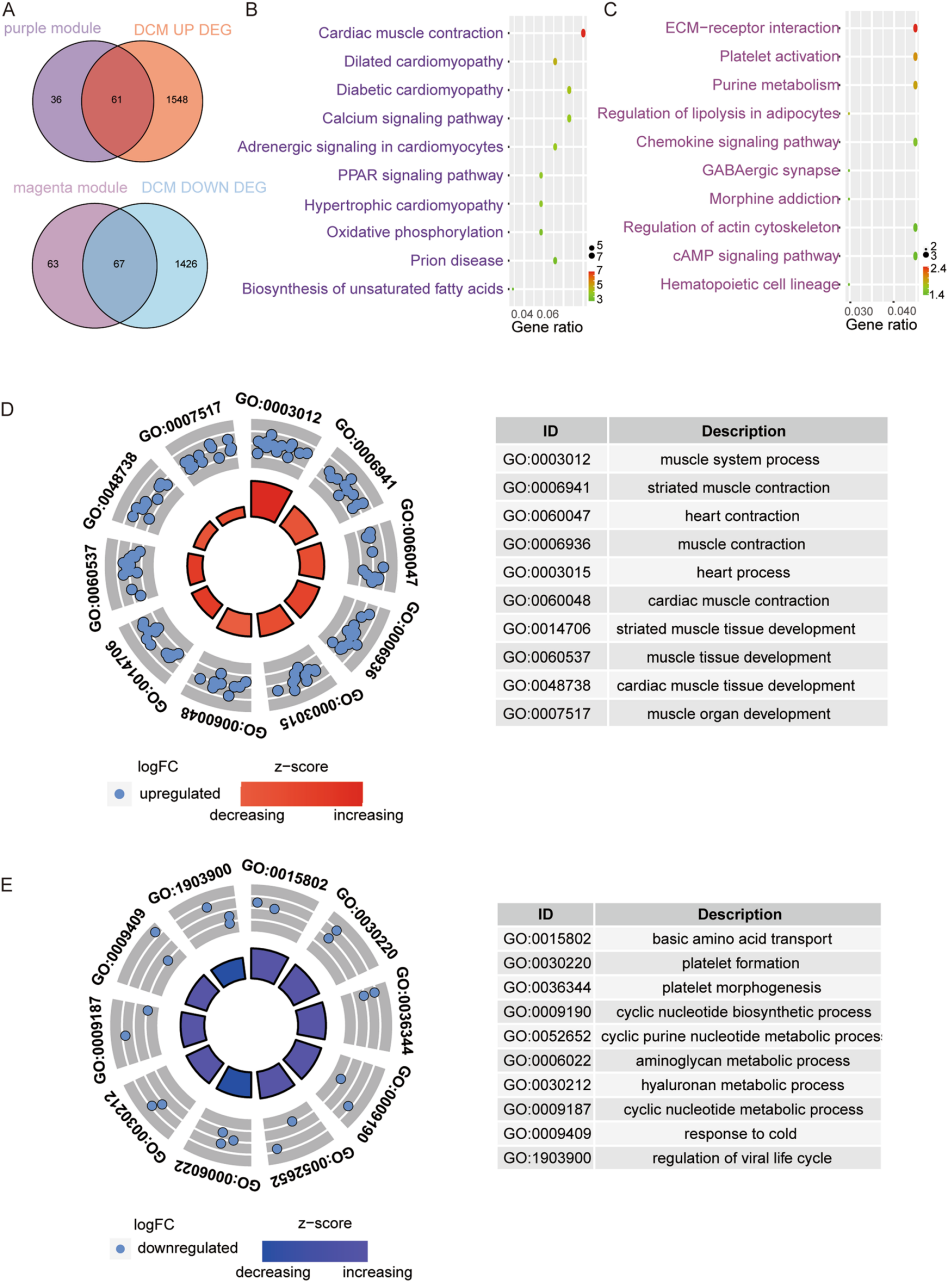
Identified Co-DEGs. (**A**) Venn diagram of common DEGs (Co-DEGs) among DEGs of the purple module (top) and the magenta module (bottom). (**B**) Results of KEGG pathway analysis on Co-DEGs based on the purple module. (**C**) Results of KEGG pathway analysis of Co-DEGs based on the magenta module. (**D**) Results of GO BP analysis of Co-DEGs based on the purple module. (**E**) Results of GO BP analysis of Co-DEGs based on the magenta module. The circles indicate the gene expression distribution in each term. Z-score value is calculated from the difference in the number of up-regulated and down-regulated genes divided by the square root of the total count.

To further explore the biological function of co-expressed DEGs (Co-DEGs), Co-DEGs were first obtained from the intersection of purple and magenta modules (Fig. 5A). Next, KEGG pathway analysis revealed that Co-DEGs in the purple module were mainly enriched in the cardiac muscle contraction pathway, while Co-DEGs in the magenta module were mainly enriched in extracellular matrix (ECM) receptor interaction pathway (Fig. 5B,C). GO Biological Process (BP) analysis found that the Co-DEGs in the purple module were mainly enriched in biological processes involved in muscle system process, and Co-DEGs in the magenta module were mainly enriched in biological processes in amino acid transportation (Fig. 5D,E).

### Hub gene identification and validation

**Figure 6:**
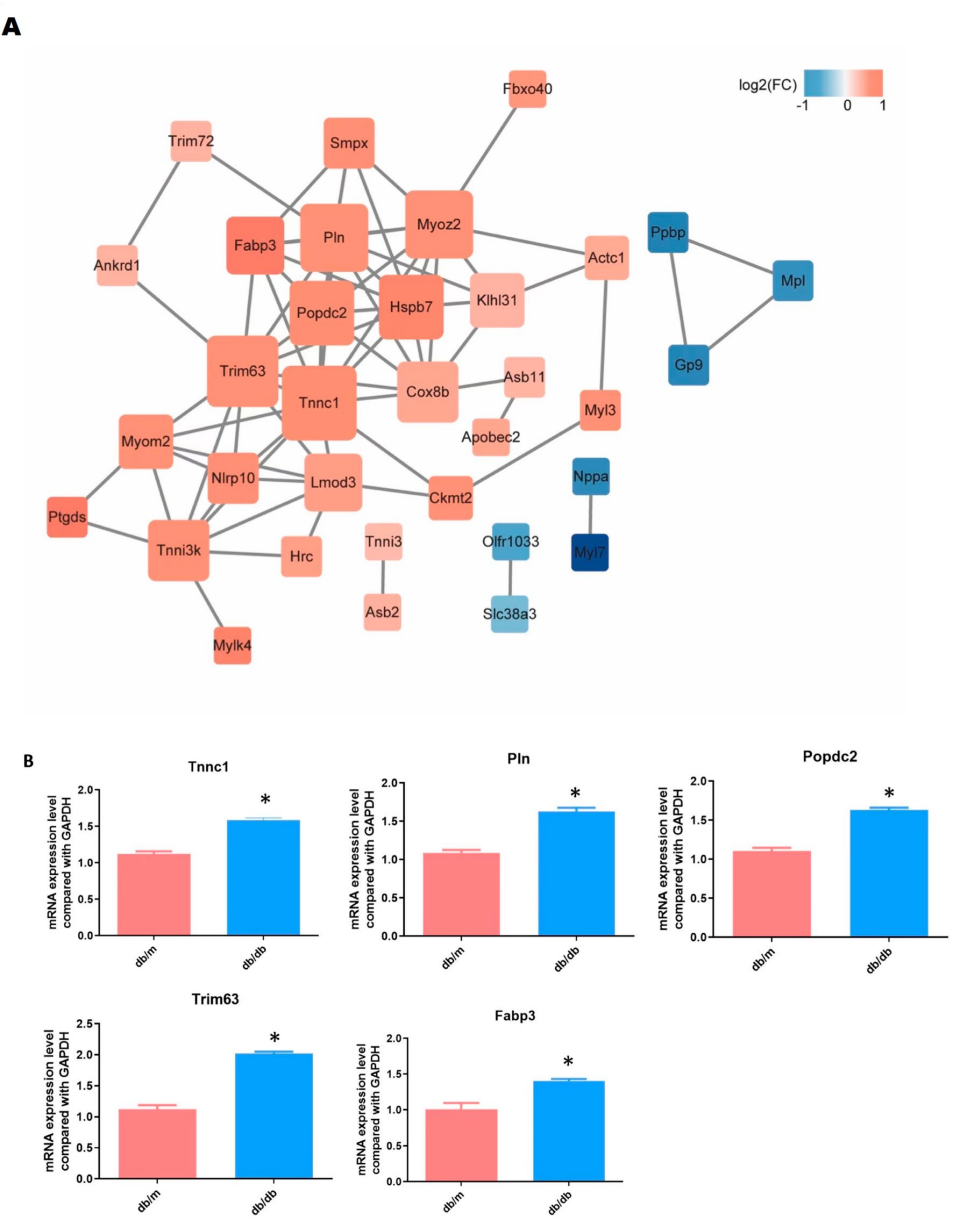
Identified and validated hub genes from co-expression network based on Co-DEGs. (**A**) Co-expression PPI network based on Co-DEGs by WGCNA. (**B**) PCR quantification of the 5 hub genes. All results are represented by at least three independent experiments. Values are presented as mean ± SD. *P ≤ 0.05.

In order to screen out the core genes in DCM, Co-DEGs in the intersection of purple and magenta modules were used to construct a co-expression network by WGCNA. The resulting data file was then processed with Cytoscape and demonstrated in Fig. 6A. In this largest connected master network, we identified 5 hub genes by cytoHubba (Table S4). Afterward, we performed real-time PCR to check the expression of the five hub genes in the cardiomyocytes derived from either db/db or db/m mice. The results (Fig. 6B) show that there were significant increases in Phospholamban (Pln), Fatty acid binding proteins 3 (Fabp3), Tripartite Motif-containing Protein 63 (Trim63), Popeye domain containing 2 (Popdc2), and Troponin C1 (Tnnc1) gene expression in DCM compared to control (P ≤ 0.05). All these results were analyzed according to bioinformatics methods.

### Prediction of target miRNAs based on hub genes

**Figure 7:**
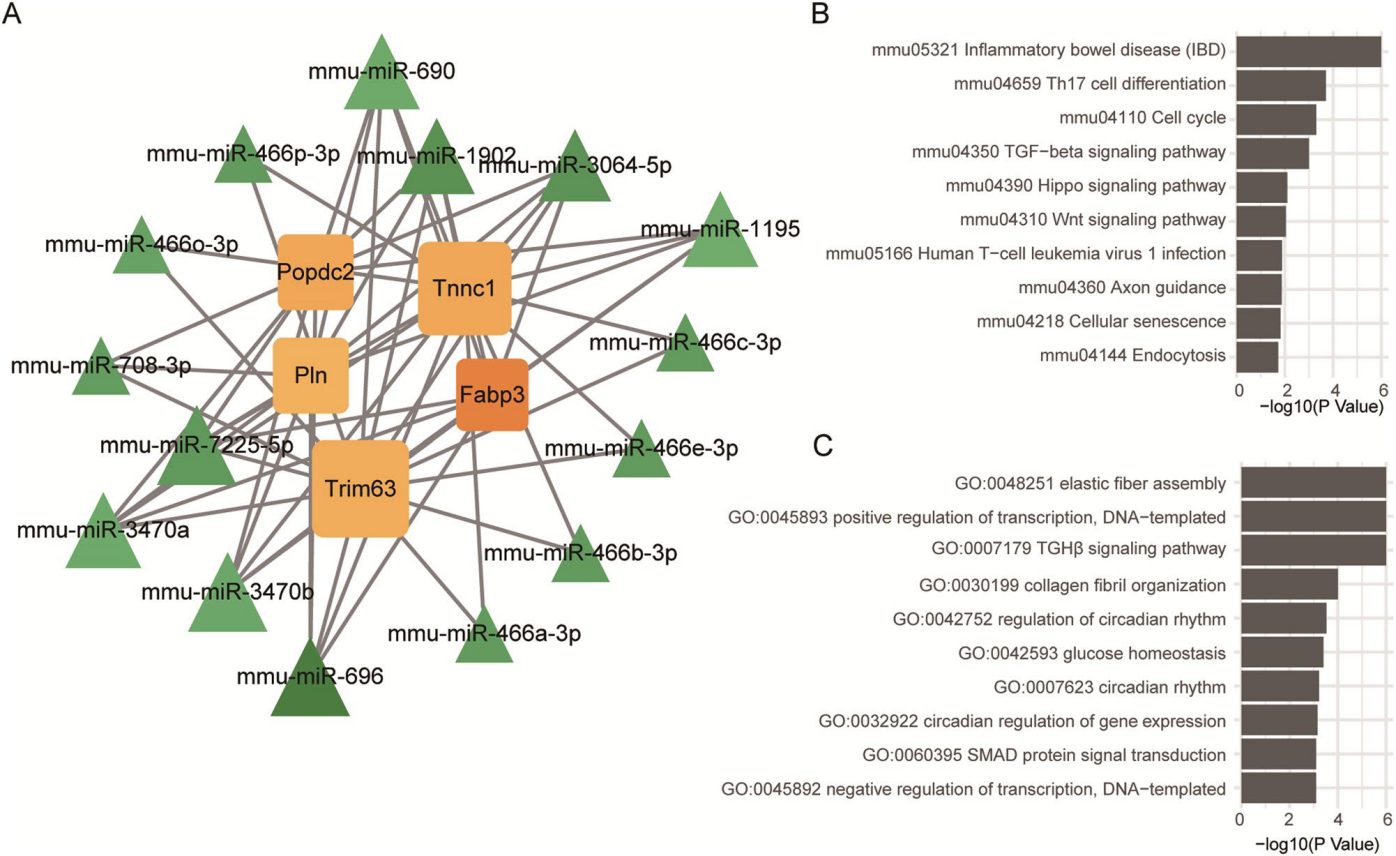
mRNA-miRNA co-expression network. (**A**) mRNA-miRNA co-expressed network constructed by Cytoscape. (**B**) Potential regulatory KEGG pathway of miRNAs from the mRNA-miRNA co-expressed network. (**C**) Potential regulatory GO BP of miRNAs from the mRNA-miRNA co-expressed network.

To further delineate the network between identified hub genes and potential target miRNAs, we used miRNA-seq to predict the target miRNAs of the hub genes mentioned above. First, we identified 52 DE-miRNAs (Fold Change ≥ 1.5, P < 0.05) in DCM (Table S5). According to these results, we predicted that the expression of hub genes was most likely regulated by miRNAs. Under this prediction, we constructed a co-expressed network between 55 hub genes and miRNAs through Cytoscape (Fig. 7A). These hub genes were linked together by shared miRNAs. Next, we performed KEGG enrichment analysis to determine the functional role of these miRNAs in the pathogenesis of DCM. The results show that the target genes of these miRNAs were mainly involved in the TGF-β signaling pathway and Wnt signaling pathway (Fig. 7B). Moreover, GO BP enrichment analysis found that these miRNAs participated in the signaling of TGF-β and glucose homeostasis (Fig. 7C). Collectively, these results suggest that these miRNAs may promote the DCM process by affecting metabolic pathways and hub gene expressions.

### Construction of CeRNA networks

**Figure 8:**
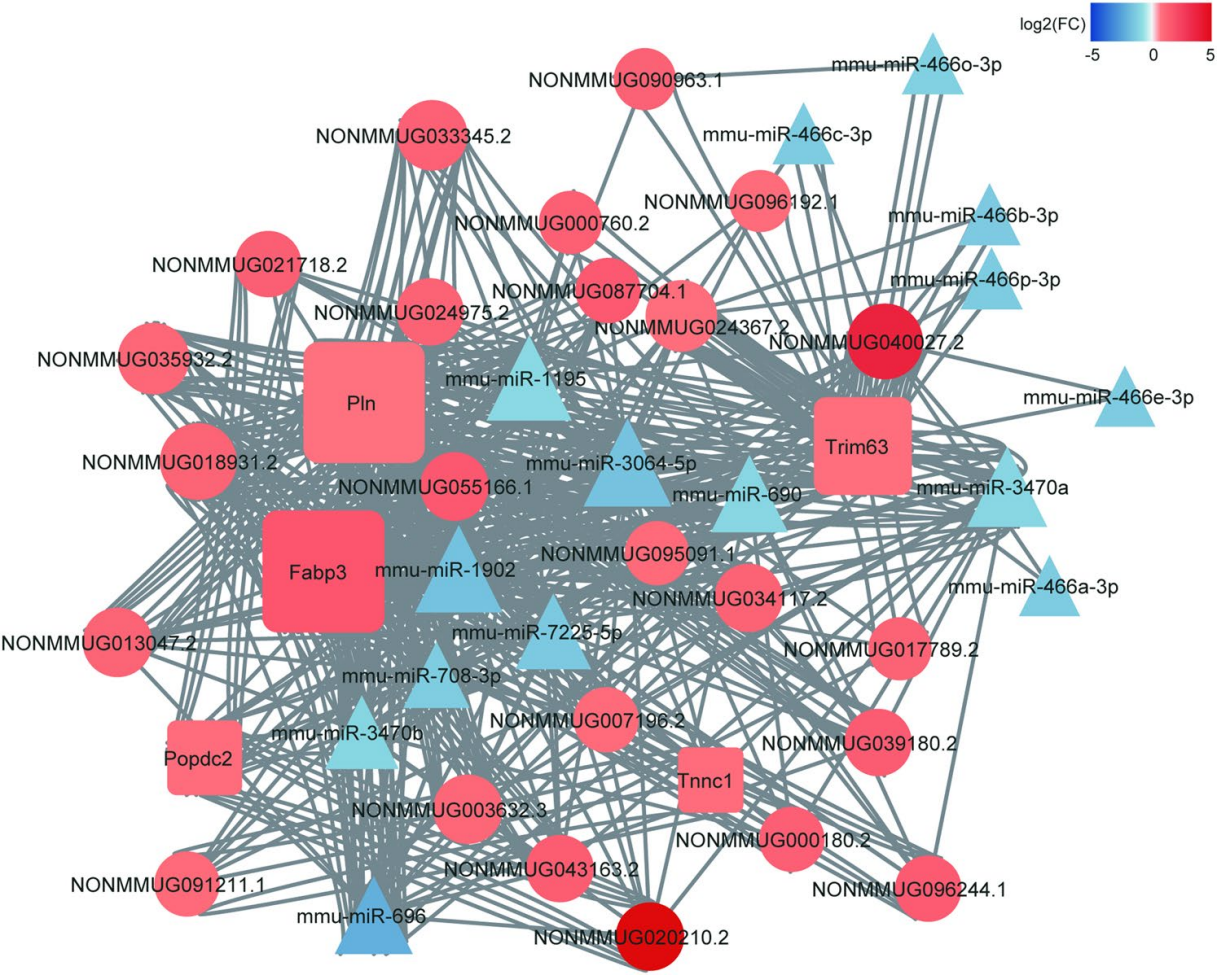
Construction of ceRNA Networks. The color intensity in each node represents the fold change of the gene in DCM, compared to control non-tumor samples (up-regulation of a gene is shown in red and down-regulation of a gene is shown in blue). The size of the circle is proportional to the score of ceRNA network.

Subsequently, according to the ceRNA hypothesis, we constructed ceRNA networks based on mRNA-miRNA co-expression network and related lncRNAs (Fig. 8). This network comprises 48 nodes and 517 edges and consists of five hub genes, 24 lncRNAs, and 15 miRNAs. Annotations of lncRNAs in ceRNA-network are shown in Table S6.

## Discussion

RNA-seq results have identified 3102 DEGS, 1135 DE-lncRNAs, and 52 DE-miRNAs between DCM mice and control. Among the DEGs, we found that up-regulated DEGs are mainly associated with oxygen metabolism, diabetes, and other pathways related to metabolic disease, consistent with previous studies^39,40^. Conversely, down-regulated DEGs are correlated to the immunity-related pathway. Recently, senolytic agents and stem cell therapy demonstrated highly promising treatments for DCM^41,42^. We found abnormal expression in an abundant of cell senescence-related genes in DCM, indicating the close association and impact of senescence on DCM^43^. Additionally, among 5 genes related to myocardial repair in mice (Hdac4, Mesp1, Ngf, Pim1, and Yap1), Ngf and Pim1 were significantly down-regulated, implying a decreased repair ability of cardiomyocytes in DCM and suggesting a potential treatment target to restore repairment in DCM.

In addition, we further performed immune cell infiltration analysis and revealed that activation of CD8^+^ T cells plays a predominant role in the development of DCM. Therefore, the development of diabetes is associated with abnormalities in the immune system. However, the role of immune cells in diabetic myocarditis is still unclear. T lymphocyte infiltration into the myocardium has been observed in a left coronary artery occlusion-induced murine myocardial infarction model and a transverse aortic constriction-induced murine pressure overload model^44^. Tang et al.^45^ reported that CD8^+^ T cells in ischemic failing human hearts may contribute to the progression of heart failure. Abdullah et al.^46^ showed that conditional T-cell sS1p1 knockout mice that exhibited sustained deficiency of both CD4^+^ and CD8^+^ T cells, had improved cardiac function and alleviated cardiac fibrosis after 11 weeks of diabetic induction, indicating that T cell Ss1p1 activation exacerbates fibrosis under hyperglycemia. Although current knowledge supports that CD4^+^ T cells play a more important role in the development of DCM, mainly via the subtype of CD4^+^Foxp3^+^ T cells^47^, we speculate that CD8^+^ T cells may be involved in the development of DCM via the following mechanisms: (1) CD8^+^ T cell can directly damage cardiomyocytes via its cytotoxicity effect; (2) CD8^+^ T cell can regulate macrophage migration via stimulating the production of nitric oxide; (3) CD8^+^ T cell can up-regulate CD11b, CD64, and CD62L on neutrophils mainly through the secretion of inflammatory factors and resultantly maintain their survival.

The current study focuses on individual diabetic complications. We analyzed the differences between tissues affected by diabetic complications by comparing them with public databases. As expected, there were differences in pathways and immune cell infiltration for each complication abnormality. We further clarified the key genes and key modules responsible for DCM by WGCNA, which were mostly differentially expressed in DCM and normally expressed in other diabetic complications. Enrichment analysis showed that up-regulation of genes related to DCM in key modules were mainly involved in calcium signaling, senescence pathways and hypertrophy related pathways, consistent with our previous comparison on DCM with DPN and DN. A recent study implementing RNA sequencing on STZ mice type 2 diabetes DCM model demonstrated that these pathways were down-regulated, which comments on the different underlying pathogenic mechanisms in type 2 diabetes, such as insulin resistance for db/db mice and islet β-cell reduction in STZ mice, that may influence DCM^48^.

Furthermore, we identified and validated 5 hub genes (Tnnc1, Pln, Fabp3, Popdc2, and Trim63) from the DCM-related key module. Mutations in Tnnc1, a complex that is known as Cardiac Troponin C and contains the component, troponin C, was confirmed to be associated with hypertrophic or dilated cardiomyopathy^49,50^. Pln is a 52-amino acid sarcoplasmic reticulum (SR) membrane protein expressed abundantly in cardiac muscle and a crucial regulator of cardiac function by modulating the rate of cardiac relaxation and size of the SR Ca2+ store^51,52^. Increased expression of Pln has been identified to reduce cardiac contractility and correlates with the over-expression of NF-SLN, raising the possibility that induced expression of SLN in human hearts can impair cardiac function^53,54^. Jia et al.^55^ also reported that the expression of Pln was significantly increased in a time-dependent manner in diabetic groups. Fatty acid-binding protein 3 (Fabp3) participates in cell metabolism by binding free long-chain fatty acids (LCFAs) and transporting them for cell metabolism^56^. Fabp3-defect exacerbates cardiac hypertrophy and heart dysfunction, but over-expression of Fabp3 can up-regulate the phosphorylation of the MAPK signaling pathway and decrease phosphorylated Akt levels, which may account for the augmentation of apoptosis and remodeling after myocardial infarction^57,58^. Popdc2, one of the Popeye domain-containing (Popdc) gene families, was highly expressed particularly in the sinoatrial node of the mouse and represented as a novel arrhythmia gene for cardiac conduction disorders^59,60^. A recent study^61^ showed that Popdc2 was a fasting-induced gene, which suggests that the abnormal expression of popdc2 may be related to blood glucose. Trim63, also known as MuRF1, was significantly increased not only in cardiac muscle of diabetic mice, but also in diabetic limb muscle and STZ-Diabetes^62,63^. Previous studies have demonstrated that abnormalities in these genes were strongly associated with the development of diabetes or cardiovascular disease. Our results classify these genes as key factors in the pathogenesis of DCM and potential drug targets for DCM treatment. However, there are only a few reports on the regulation of these genes with miRNA or ceRNA, except Trim63^64^.

In this study, we first reported the potential regulatory network among DCM hub genes, DCM-related miRNAs, and ceRNA networks. Most of these miRNAs, such as miR-3064-5p^65^, miR-690^66^, miR-1195^67,68^, miR-696^69,70^, miR-708-3p^71^, miR-7225-5p^72^, miR-466 (including miR-466b-3p, miR-466c-3p, miR-466p-3p, miR-466a-3p, miR-466e-3p, miR-466o-3p)^73^, have been reported to be involved in diabetes or cardiovascular disease. This supports our speculation that these miRNAs may play an important role in DCM. On the other hand, most lncRNAs in the ceRNA network have yet identified to be participating in cardiovascular pathology. More in-depth research will be worth conducting to address the involvement of lncRNAs.

## Conclusion

Overall, we systematically analyzed the characteristics of both mRNA and noncoding expression profiles in DCM. We identified the potential mechanisms and the hub genes in DCM pathogenesis, namely Phospholamban (Pln), Fatty acid binding proteins 3 (Fabp3), Tripartite Motif-containing Protein 63 (Trim63), Popeye domain containing 2 (Popdc2), and Troponin C1 (Tnnc1). Additionally, we also found potential mRNA-miRNA and ceRNA networks in DCM. Our results shed light on further studies of DCM pathogenesis and on the discovery of DCM therapeutic targets.

## Supplementary Information

Supplementary Information 1.

Supplementary Information 2.

Supplementary Information 3.

Supplementary Information 4.

Supplementary Information 5.

Supplementary Information 6.

Supplementary Information 7.

Supplementary Information 8.

Supplementary Information 9.

Supplementary Information 10.

## Data Availability

The datasets generated and/or analyzed during the current study are available in the GEO datasets repository under accession GSE211108 [https://www.ncbi.nlm.nih.gov/geo/query/acc.cgi?acc=GSE211108], GSE211107 [https://www.ncbi.nlm.nih.gov/geo/query/acc.cgi?acc=GSE211107], and GSE211106 [https://www.ncbi.nlm.nih.gov/geo/query/acc.cgi?acc=GSE211106].

## References

[1] Lee MMY, McMurray JJV, Lorenzo-Almorós A, Kristensen SL, Sattar N, Jhund PS (2019). Diabetic cardiomyopathy. Heart.

[2] Borghetti G, Von Lewinski D, Eaton DM, Sourij H, Houser SR, Wallner M (2018). Diabetic cardiomyopathy: Current and future therapies. Beyond glycemic control. Front. Physiol..

[3] Lorenzo-Almorós A, Tuñón J, Orejas M, Cortés M, Egido J, Lorenzo O (2017). Diagnostic approaches for diabetic cardiomyopathy. Cardiovasc. Diabetol..

[4] Gulsin GS, Athithan L, McCann GP (2019). Diabetic cardiomyopathy: Prevalence, determinants and potential treatments. Ther. Adv. Endocrinol..

[5] Fernandes JCR, Acuña SM, Aoki JI, Floeter-Winter LM, Muxel SM (2019). Long non-coding RNAs in the regulation of gene expression: Physiology and disease. Noncoding RNA.

[6] Palazzo AF, Lee ES (2015). Non-coding RNA: What is functional and what is junk?. Front. Gene.

[7] Filardi T, Catanzaro G, Mardente S, Zicari A, Santangelo C, Lenzi A, Morano S, Ferretti E (2020). Non-coding RNA: Role in gestational diabetes pathophysiology and complications. Int. Mol. Sci..

[8] He J, Li X, Zhang Y, Zhang Q, Li L (2021). Comprehensive analysis of ceRNA regulation network involved in the development of coronary artery disease. BioMed. Res. I.

[9] Qi X, Zhang DH, Wu N, Xiao JH, Wang X, Ma W (2015). ceRNA in cancer: Possible functions and clinical implications. J. Med. Gene.

[10] Zhou RS, Zhang EX, Sun QF, Ye ZJ, Liu JW, Zhou DH, Tang Y (2019). Integrated analysis of lncRNA-miRNA-mRNA ceRNA network in squamous cell carcinoma of tongue. BMC Cancer.

[11] Ma N, Tie C, Yu B, Zhang W, Wan J (2020). Identifying lncRNA-miRNA-mRNA networks to investigate Alzheimer’s disease pathogenesis and therapy strategy. Aging.

[12] Zhou X, Zhang W, Jin M, Chen J, Xu W, Kong X (2017). lncRNA MIAT functions as a competing endogenous RNA to upregulate DAPK2 by sponging miR-22-3p in diabetic cardiomyopathy. Cell Death Dis..

[13] Feng Y, Xu W, Zhang W, Wang W, Liu T, Zhou X (2019). LncRNA DCRF regulates cardiomyocyte autophagy by targeting miR-551b-5p in diabetic cardiomyopathy. Theranostics.

[14] Yang F, Qin Y, Lv J, Wang Y, Che H, Chen X (2018). Silencing long non-coding RNA Kcnq1ot1 alleviates pyroptosis and fibrosis in diabetic cardiomyopathy. Cell. Death Dis..

[15] Ni T, Huang X, Pan S, Lu Z (2021). Inhibition of the long non-coding RNA ZFAS1 attenuates ferroptosis by sponging miR-150-5p and activates CCND2 against diabetic cardiomyopathy. J. Cell. Mol. Med..

[16] Hou J, Zheng D, Zhong G, Hu Y (2013). Mangiferin mitigates diabetic cardiomyopathy in streptozotocin-diabetic rats. Can. J. Physiol. Pharmacol..

[17] Hinder LM, Park M, Rumora AE, Hur J, Eichinger F, Pennathur S (2017). Comparative RNA-Seq transcriptome analyses reveal distinct metabolic pathways in diabetic nerve and kidney disease. J. Cell. Mol. Med..

[18] Abouelkhair MA (2020). Non-SARS-CoV-2 genome sequences identified in clinical samples from COVID-19 infected patients: Evidence for co-infections. PeerJ.

[19] Kim T, Seo HD, Hennighausen L, Lee D, Kang K (2018). Octopus-toolkit: A workflow to automate mining of public epigenomic and transcriptomic next-generation sequencing data. Nucleic Acids Res..

[20] Zhao L, Wang J, Li Y, Song T, Wu Y, Fang S (2021). NONCODEV6: An updated database dedicated to long non-coding RNA annotation in both animals and plants. Nucleic Acids Res..

[21] Frankish A, Diekhans M, Jungreis I, Lagarde J, Loveland JE, Mudge JM (2021). GENCODE 2021. Nucleic Acids Res..

[22] Kim J, Lim H, Moon B, Ang M, Kim S, Moon C (2021). Epigenetic mechanisms involved in the neuroprotective effect of scorpion extract in a Parkinson's disease murine model based on multi-omics approach. J. Tradit. Chin. Med..

[23] Kalvari I, Nawrocki EP, Ontiveros-Palacios N, Argasinska J, Lamkiewicz K, Marz M, Griffiths-Jones S, Toffano-Nioche C, Gautheret D, Weinberg Z (2021). Rfam 14: Expanded coverage of metagenomic, viral and microRNA families. Nucleic Acids Res..

[24] Friedländer MR, MacKowiak SD, Li N, Chen W, Rajewsky N (2012). MiRDeep2 accurately identifies known and hundreds of novel microRNA genes in seven animal clades. Nucleic Acids Res..

[25] Zhang B, Horvath S (2005). A general framework for weighted gene co-expression network analysis. Stat. Appl. Genet. Mol. Biol..

[26] Qin Q, Fang DL, Zhou W, Meng Y, Wei J (2021). Classification and immune invasion analysis of breast cancer based on m6A genes. Ann. Transl. Med..

[27] Deng X, Bi Q, Chen S, Chen X, Li S, Zhong Z (2021). Identification of a five-autophagy-related-lncRNA signature as a novel prognostic biomarker for hepatocellular carcinoma. Front. Mol. Biosci..

[28] Kanehisa M, Sato Y, Kawashima M, Furumichi M, Tanabe M (2016). KEGG as a reference resource for gene and protein annotation. Nucleic Acids Res..

[29] Kanehisa M, Goto S (2020). KEGG: Kyoto encyclopedia of genes and genomes. Nucleic Acids Res..

[30] Yu G, Wang LG, Han Y, He QY (2012). ClusterProfiler: An R package for comparing biological themes among gene clusters. OMICS.

[31] Subramanian A, Tamayo P, Mootha VK, Mukherjee S, Ebert BL, Gillette MA (2005). Gene set enrichment analysis: A knowledge-based approach for interpreting genome-wide expression profiles. PNAS.

[32] Zhao M, Chen L, Qu H (2016). CSGene: A literature-based database for cell senescence genes and its application to identify critical cell aging pathways and associated diseases. Cell Death Dis..

[33] Kang W, Jin T, Zhang T, Ma S, Yan H, Liu Z, Ji Z, Cai Y, Wang S, Song M, Ren J, Hu B, Zhou Q, Zhang W, Qu J, Bao Y, Liu GH (2022). Regeneration Roadmap: Database resources for regenerative biology. Nucleic Acids Res..

[34] Xiao B, Liu L, Li A, Xiang C, Wang P, Li H, Xiao T (2020). Identification and verification of immune-related gene prognostic signature based on ssGSEA for osteosarcoma. Front. Oncol..

[35] Zhang L, Zhao Y, Dai Y, Cheng JN, Gong Z, Feng Y (2018). Immune landscape of colorectal cancer tumor microenvironment from different primary tumor location. Front. Immunol..

[36] Chen Z, Huang A, Sun J, Jiang T, Qin FXF, Wu A (2017). Corrigendum: Inference of immune cell composition on the expression profiles of mouse tissue. Sci. Rep..

[37] Kang JQ, Song YX, Liu L, Lu Y, Tian J, Hu R, Wang X, Liu XQ (2021). Identification of key genes in type 2 diabetes-induced erectile dysfunction rats with stem cell therapy through high-throughput sequencing and bioinformatic analysis. Andrologia.

[38] Zhang Q, Wang JY, Zhou SY, Yang SJ, Zhong SL (2019). Circular RNA expression in pancreatic ductal adenocarcinoma. Oncol. Lett..

[39] Isfort M, Stevens SCW, Schaffer S, Jong CJ, Wold LE (2014). Metabolic dysfunction in diabetic cardiomyopathy. Heart Fail. Rev..

[40] Battiprolu PK, Lopez-Crisosto C, Wang ZV, Nemchenko A, Lavandero S, Hill JA (2013). Diabetic cardiomyopathy and metabolic remodeling of the heart. Life Sci..

[41] Salerno N, Marino F, Scalise M, Salerno L, Molinaro C, Filardo A (2022). Pharmacological clearance of senescent cells improves cardiac remodeling and function after myocardial infarction in female aged mice. Mech. Ageing Dev..

[42] Salerno N, Salerno L, Marino F, Scalise M, Chiefalo A, Panuccio G (2022). Myocardial regeneration protocols towards the routine clinical scenario: An unseemly path from bench to bedside. EClinicalMedicine.

[43] Marino F, Scalise M, Salerno N, Salerno L, Molinaro C, Cappetta D (2022). Diabetes-induced cellular senescence and senescence-associated secretoryphenotype impair cardiac regeneration and function independently of age. Diabetes.

[44] Hofmann U, Frantz S (2015). Role of lymphocytes in myocardial injury, healing, and remodeling after myocardial infarction. Circ. Res..

[45] Tang TT, Zhu YC, Dong NG, Zhang S, Cai J, Zhang LX (2019). Pathologic T-cell response in ischaemic failing hearts elucidated by T-cell receptor sequencing and phenotypic characterization. Eur. Heart J..

[46] Abdullah CS, Jin ZQ (2018). Targeted deletion of T-cell S1P receptor 1 ameliorates cardiac fibrosis in streptozotocin-induced diabetic mice. Faseb J..

[47] Abdullah CS, Li Z, Wang X, Jin ZQ (2016). Depletion of T lymphocytes ameliorates cardiac fibrosis in streptozotocin-induced diabetic cardiomyopathy. Int. Immunopharmacol..

[48] Marino F, Salerno N, Scalise M, Salerno L, Torella A, Molinaro C (2023). Streptozotocin-induced type 1 and 2 diabetes mellitus mouse models show different functional, cellular and molecular patterns of diabetic cardiomyopathy. Int. J. Mol. Sci..

[49] Li MX, Hwang PM (2015). Structure and function of cardiac troponin C (TNNC1): Implications for heart failure, cardiomyopathies, and troponin modulating drugs. Gene.

[50] Landstrom AP, Parvatiyar MS, Pinto JR, Marquardt ML, Bos JM, Tester DJ (2008). Molecular and functional characterization of novel hypertrophic cardiomyopathy susceptibility mutations in TNNC1-encoded troponin C. J. Mol. Cell. Cardiol..

[51] Tada M, Kadoma M (1989). Regulation of the Ca2+ pump atpase by cAMP-dependent phosphorylation of phospholamban. BioEssays.

[52] Kiriazis H, Kranias EG (2000). Genetically engineered models with alterations in cardiac membrane calcium-handling proteins. Annu. Rev. Physiol..

[53] Kadambi VJ, Ponniah S, Harrer JM, Hoit BD, Dorn GW, Walsh RA (1996). Cardiac-specific overexpression of phospholamban alters calcium kinetics and resultant cardiomyocyte mechanics in transgenic mice. J. Clin. Investig..

[54] Asahi M, Otsu K, Nakayama H, Hikoso S, Takeda T, Gramolini AO (2004). Cardiac-specific overexpression of sarcolipin inhibits sarco(endo)plasmic reticulum Ca2+ ATPase (SERCA2a) activity and impairs cardiac function in mice. PNAS.

[55] Jia Z, Sun J, Li HZ, Li HX, Peng X, Shao HJ (2015). Decreased expression of calcium-sensing receptor involved in the progression of diabetic cardiomyopathy. Zhongguo Ying Yong Sheng Li Xue Za Zhi.

[56] Lee SM, Lee SH, Jung Y, Lee Y, Yoon JH, Choi JY (2020). FABP3-mediated membrane lipid saturation alters fluidity and induces ER stress in skeletal muscle with aging. Nat. Commun..

[57] Zhuang L, Li C, Chen Q, Jin Q, Wu L, Lu L (2019). Fatty acid-binding protein 3 contributes to ischemic heart injury by regulating cardiac myocyte apoptosis and MAPK pathways. Am. J. Physio-Heart Circ. Physiol..

[58] Zhuang L, Mao Y, Liu Z, Li C, Jin Q, Lu L (2021). FABP3 deficiency exacerbates metabolic derangement in cardiac hypertrophy and heart failure via PPARα pathway. Front. Cardiovasc. Med..

[59] Froese A, Brand T (2008). Expression pattern of Popdc2 during mouse embryogenesis and in the adult. Dev. Dyn..

[60] Rinné S, Ortiz-Bonnin B, Stallmeyer B, Kiper AK, Fortmüller L, Schindler RFR (2020). POPDC2 a novel susceptibility gene for conduction disorders. J. Mol. Cell. Cardiol..

[61] Defour M, Michielsen CCJR, O’donovan SD, Afman LA, Kersten S (2020). Transcriptomic signature of fasting in human adipose tissue. Physiol. Genom..

[62] van Luteren E, Moyer M (2009). Gene expression profiling in the type 1 diabetes rat diaphragm. PLoS ONE.

[63] O’Neill BT, Bhardwaj G, Penniman CM, Krumpoch MT, Suarez Beltran PA, Klaus K (2019). FOXO transcription factors are critical regulators of diabetes-related muscle atrophy. Diabetes.

[64] Gerlinger-Romero F, Yonamine CY, Junior DCP, Esteves JVDC, Machado UF (2017). Dysregulation between TRIM63/FBXO32 expression and soleus muscle wasting in diabetic rats: Potential role of miR-1-3p, -29a/b-3p, and -133a/b-3p. Mol. Cell. Biochem..

[65] Yang W, Tu H, Tang K, Huang H, Ou S, Wu J (2021). MiR-3064 in epicardial adipose-derived exosomes targets neuronatin to regulate adipogenic differentiation of epicardial adipose stem cells. Front. Cardiovasc. Med..

[66] Kim MZX (2019). The profiling and role of miRNAs in diabetes mellitus. J. Diabetes Clin. Res..

[67] Humphreys DT, Hynes CJ, Patel HR, Wei GH, Cannon L, Fatkin D (2012). Complexity of murine cardiomyocyte miRNA biogenesis, sequence variant expression and function. PLoS ONE.

[68] Xue J, Zhou D, Poulsen O, Hartley I, Imamura T, Xie EX (2018). Exploring miRNA-mRNA regulatory network in cardiac pathology in Na+/H+ exchanger isoform 1 transgenic mice. Physiol. Genom..

[69] Fang Z, Li P, Jia W, Jiang T, Wang Z, Xiang Y (2016). MiR-696 plays a role in hepatic gluconeogenesis in ob/ob mice by targeting PGC-1?. Int. J. Mol. Med..

[70] Aoi W, Naito Y, Mizushima K, Takanami Y, Kawai Y, Ichikawa H (2010). The microRNA miR-696 regulates PGC-1α in mouse skeletal muscle in response to physical activity. Am. J. Physiol. Endocrinol. Metab..

[71] Qu Y, Zhang J, Zhang J, Xiao W (2021). MiR-708-3p alleviates inflammation and myocardial injury after myocardial infarction by suppressing ADAM17 expression. Inflammation.

[72] Wang X, Song C, Zhou X, Han X, Li J, Wang Z (2017). Mitochondria associated MicroRNA expression profiling of heart failure. BioMed. Res. Int..

[73] Liu H, Chen X, Liang M, Qin H, Rong J, Yao JP (2014). Atrial fibrillation alters the microRNA expression profiles of the left atria of patients with mitral stenosis. BMC Cardiovasc. Disord..

