# Supplementary figures and images for: Identification of hub genes and potential ceRNA networks of diabetic cardiomyopathy
Source: Sci Rep. 2023 Jun 24;13:10258. doi: 10.1038/s41598-023-37378-5 (PMC10290640; doi:10.1038/s41598-023-37378-5)

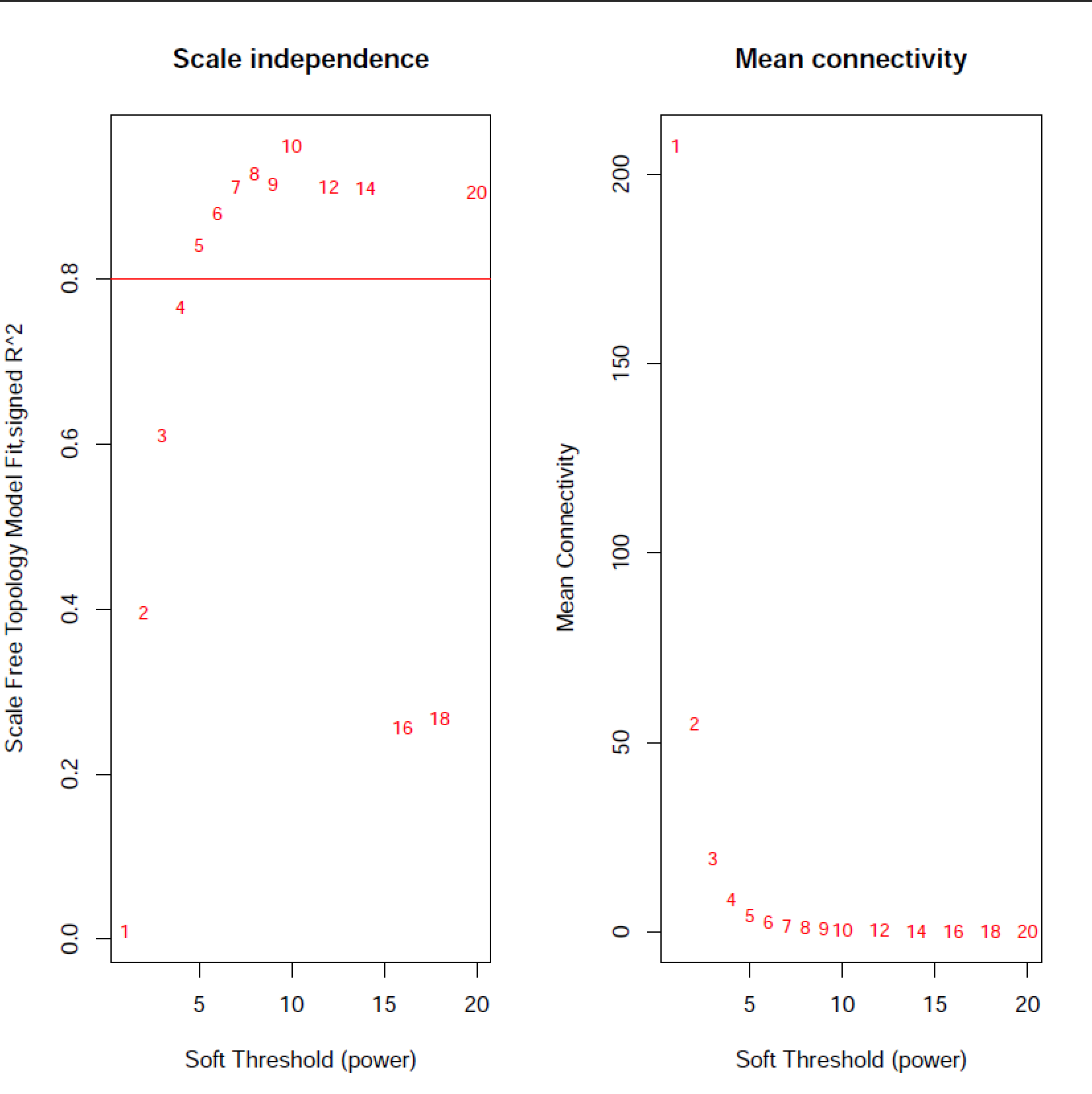

Supplement: Supplementary file 1 — Supplementary Information 1. [file 41598_2023_37378_MOESM1_ESM.tif]

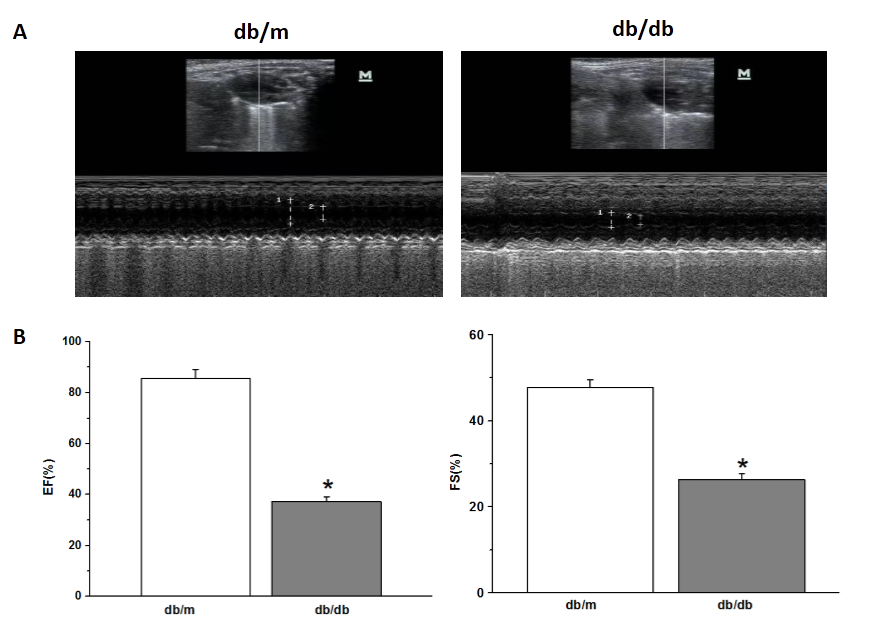

Supplement: Supplementary file 2 — Supplementary Information 2. [file 41598_2023_37378_MOESM2_ESM.tif]

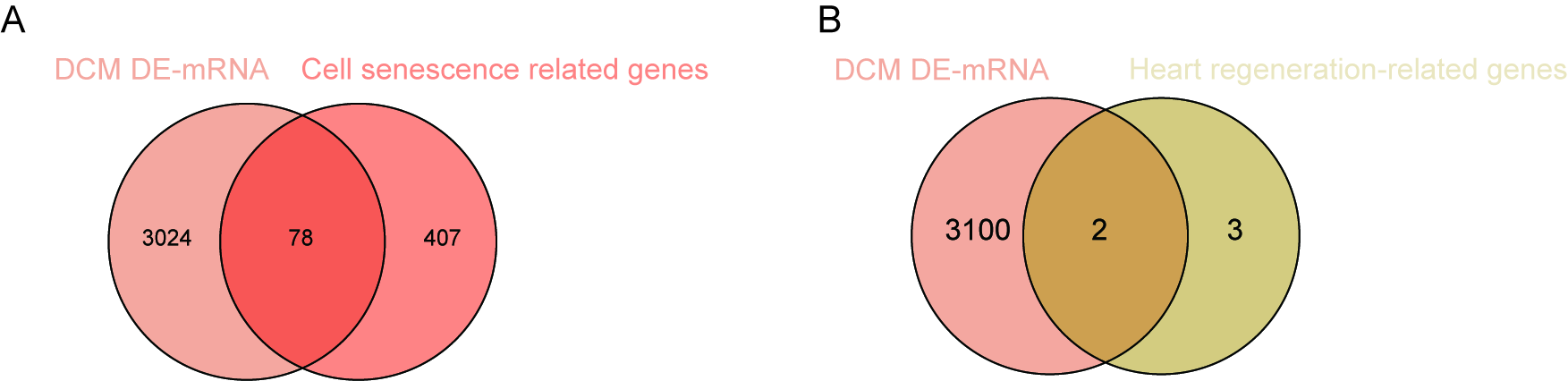

Supplement: Supplementary file 3 — Supplementary Information 3. [file 41598_2023_37378_MOESM3_ESM.tif]
