# Supplementary material for: Identification of hub genes and potential ceRNA networks of diabetic cardiomyopathy
Source: Sci Rep. 2023 Jun 24;13:10258. doi: 10.1038/s41598-023-37378-5 (PMC10290640; doi:10.1038/s41598-023-37378-5)
Supplement: Supplementary file 5 — Supplementary Information 5. [file 41598_2023_37378_MOESM5_ESM.docx]

**Table S2. The primers sequencing for qRT-PCR**

| Tnnc1-F | AGCTGCGGTAGAACAGTTGA |
| --- | --- |
| TnnC1-R | GCTCCTTGGTGCTGATGC |
| Pln-F | GAAGCCAAGACAGAAGCAGG |
| Pln-R | TGACAACAGGCAGCCAAAT |
| Popdc2-F | GACTGCGGAGACCGAATGT |
| Popdc2-R | CTGAGAAGAGGCGGGAGAT |
| Trim63-F | ATGGAAACGCTATGGAGAACC |
| Trim63-R | TTGGCAGGGCAGGATGA |
| Fabp3-F | CGGACTTATGAGAAGGAGGC |
| Fabp3-R | AATGTCAGAGGGGAAAACCA |
| m-GAPDH-S | TGAAGCAGGCATCTGAGGG |
| m-GAPDH-A | TGAAGTCGCAGGAGACAACC |
