# Supplementary material for: Identification of hub genes and potential ceRNA networks of diabetic cardiomyopathy
Source: Sci Rep. 2023 Jun 24;13:10258. doi: 10.1038/s41598-023-37378-5 (PMC10290640; doi:10.1038/s41598-023-37378-5)
Supplement: Supplementary file 10 — Supplementary Information 10. [file 41598_2023_37378_MOESM10_ESM.rtf]

SUPPLEMENTARY FIGURE AND TABLE LEGENDS

Figure S1. The effect of different power values on the scale independence degree and mean connectivity of co-expression modules. Left: the X-axis reflects the soft-thresholding power and Y-axis reflects the scale-free topology model fit index. Right: the X-axis reflects the soft-thresholding power and Y-axis reflects the mean connectivity.
Figure S2. DCM model evaluation. A) Representative echocardiographic image. B) EF and FS quantification. All results are representative of at least three independent experiments. Values are presented as mean ± SD. *, p ≤ 0.05.
Figure S3. Differential expression cell senescence related genes and heart regeneration-related genes. A) Venn diagram showed the intersection of DE-mRNAs and cell senescence related genes. B) Venn diagram showed the intersection of DE-mRNAs and heart regeneration-related genes. 
Table S1. The 25 immune cells related signature.
Table S2. The primers sequences for qRT-PCR.
Table S3. DE-mRNAs and DE-lncRNAs.
Table S4. The detailed information of 5 hub genes 
Table S5. DE-miRNAs.
Table S6. The annotation of lncRNAs in ceRNA-network.
